# Huoxue Jiegu compound capsule accelerates tibial fracture healing via angiogenesis-driven repair mechanisms

**DOI:** 10.3389/fmed.2026.1810673

**Published:** 2026-05-11

**Authors:** Weisong Lu, Bin Pu, Dong Wang, Shixiang Tan, Yingjie Wu, Yangzhan Sun, Mengze Li, Hegui Xu

**Affiliations:** 1 Guizhou University of Traditional Chinese Medicine, Guiyang, Guizhou, China; 2 Department of Orthopedics and Traumatology I, Luzhou Hospital of Traditional Chinese Medicine, Luzhou, Sichuan, China; 3 Department of Orthopedics and Traumatology I, Suining Hospital of Traditional Chinese Medicine, Suining, Sichuan, China; 4 Department of Orthopaedics, Guizhou Provincial Third People's Hospital, Guiyang, Guizhou, China

**Keywords:** angiogenesis, fracture healing, Huoxue Jiegu compound capsule, molecular dynamics simulation, network pharmacology, neuro–vascular–bone interaction, tibial fracture repair

## Abstract

**Background:**

Fracture healing is a complex regenerative process requiring coordinated interactions among vascular, skeletal, and neural systems. Angiogenesis is a critical rate-limiting step that regulates oxygen delivery, inflammatory resolution, and osteogenic cell recruitment within the fracture microenvironment. Huoxue Jiegu Compound Capsule (HXJGCC), a traditional Chinese medicine formulation, has demonstrated clinical efficacy in fracture management; however, its molecular mechanisms underlying fracture repair remain insufficiently elucidated.

**Methods:**

An integrative strategy combining network pharmacology, molecular docking, 100-ns molecular dynamics simulations, and *in vivo* experimental validation was employed to investigate the mechanisms of HXJGCC in tibial fracture healing. Candidate bioactive compounds and potential therapeutic targets were identified, followed by protein–protein interaction (PPI) network construction and functional enrichment analyses. A rabbit tibial segmental defect model was established to validate angiogenesis-related effects using histological analysis and qRT-PCR.

**Results:**

A total of 209 candidate active compounds and 185 overlapping targets were identified. Network analysis revealed key hub targets, including AKT1, STAT3, IL6, BCL2, EGFR, and JUN, which were significantly enriched in angiogenesis-related signaling pathways, particularly HIF-1, PI3K–Akt, Relaxin, TNF, and FoxO pathways. Molecular docking and molecular dynamics simulations demonstrated stable interactions between core compounds and target proteins, supporting a multi-component and multi-target mechanism. In vivo experiments showed that HXJGCC significantly increased vascular density and improved vascular architecture in the fracture region. qRT-PCR analysis further confirmed significant upregulation of angiogenesis-associated genes (AKT1, STAT3, IL6, and EGFR), indicating activation of pro-angiogenic regulatory networks.

**Conclusion:**

HXJGCC accelerates tibial fracture healing primarily by enhancing angiogenesis and improving the local microvascular microenvironment. Mechanistically, activation of HIF-1/PI3K–Akt-related signaling pathways may coordinate inflammatory responses, vascular remodeling, and downstream osteogenic processes. These findings support the establishment of an angiogenesis-driven repair framework, potentially integrating neuro–vascular–bone interactions, and provide mechanistic insights into the multi-target therapeutic effects of HXJGCC, highlighting angiogenesis-centered regulation as a promising strategy for promoting bone regeneration.

## Introduction

Efficient fracture healing depends on the coordinated interaction of multiple biological systems and remains a major clinical challenge, particularly in cases of delayed union or nonunion (1). At the tissue level, bone repair proceeds through a sequence of highly regulated biological phases, including inflammation, angiogenesis, osteogenic differentiation, and callus remodeling (2). Among these processes, angiogenesis is widely recognized as a critical determinant of successful fracture repair, as newly formed blood vessels provide oxygen, nutrients, and progenitor cell recruitment while simultaneously delivering angiocrine signals that regulate osteoblast differentiation and bone microenvironment remodeling (3). Disruption of vascular formation significantly compromises bone regeneration, highlighting the fundamental role of vascular–bone coupling in skeletal repair (4).

Recent advances in skeletal biology have further emphasized that the fracture microenvironment is regulated by complex interactions among multiple tissue systems. In addition to vascular regeneration, emerging evidence suggests that sensory nerve signaling may participate in the regulation of bone repair by influencing inflammatory responses, endothelial activation, and osteogenic activity (5). Bone tissue is richly innervated by sensory nerve fibers that become rapidly activated after fracture (6). These nerves release various neuropeptides and neurotrophic factors that have been reported to modulate vascular growth and bone formation. Experimental studies have shown that disruption of sensory nerve signaling may impair callus formation and delay bone remodeling, suggesting that neural inputs may influence the regenerative microenvironment during fracture healing (7). Collectively, these findings indicate that fracture repair may involve coordinated interactions among neural, vascular, and skeletal systems rather than a single regulatory axis (8).

From this perspective, fracture healing can be viewed as a multifactorial regenerative process dominated by angiogenesis but potentially coordinated with neural and osteogenic signaling pathways. Angiogenesis plays a central role in establishing a supportive microenvironment that enables subsequent bone formation and tissue remodeling, while neural signaling may contribute to the regulation of local cellular responses and vascular activity. Therefore, therapeutic strategies capable of enhancing vascular regeneration while simultaneously modulating related biological networks may provide effective approaches for improving fracture healing outcomes (9, 10).

Traditional Chinese medicine (TCM) traumatology provides a systemic framework for understanding fracture repair. According to classical TCM theory, traumatic injury leads to obstruction of qi and blood circulation within meridians and bone channels, thereby impairing tissue nourishment and regeneration (11, 12). Therapeutic strategies therefore emphasize promoting blood circulation, removing blood stasis, and reconnecting bone and tendons, which are believed to restore the local regenerative microenvironment and accelerate bone healing (13). Increasing pharmacological evidence suggests that many TCM formulations used for fracture management exert biological activities related to angiogenesis, anti-inflammatory regulation, and osteogenic stimulation, supporting their potential role in modern bone regeneration therapy.

HXJGCC is a classical TCM prescription developed based on these therapeutic principles and has been widely applied clinically for the treatment of fractures and delayed bone healing (14, 15). Although its clinical efficacy has been recognized, the underlying molecular mechanisms remain incompletely understood, particularly regarding how its multiple bioactive components may regulate vascular regeneration and fracture repair processes. Considering the central role of angiogenesis in bone healing, it is reasonable to hypothesize that HXJGCC may promote fracture repair by improving the local vascular microenvironment and activating key signaling pathways associated with tissue regeneration.

**Figure 1:**
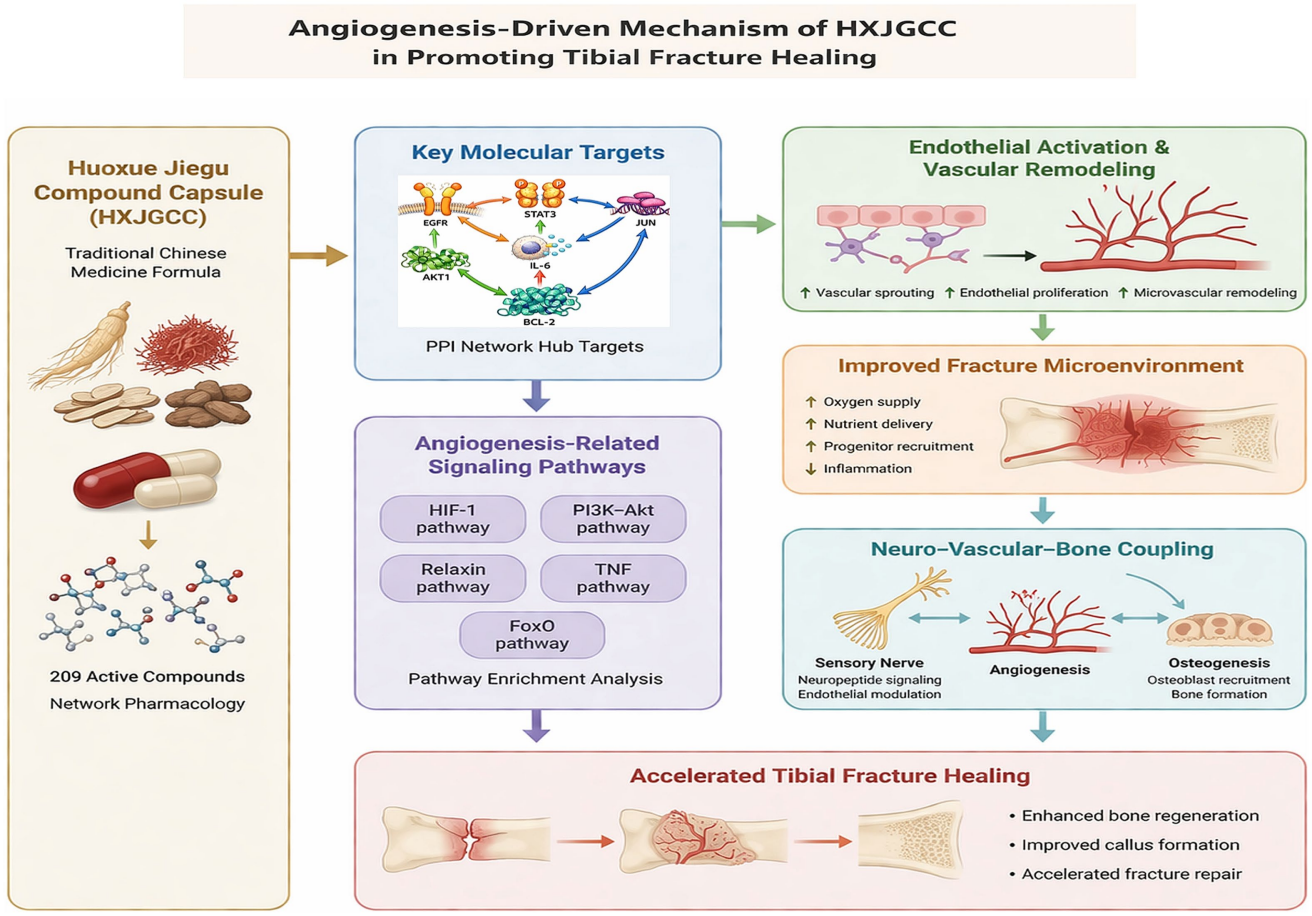
Schematic illustration of the proposed mechanism by which HXJGCC promotes tibial fracture healing through angiogenesis-driven repair mechanisms and potential neuro–vascular–bone interactions.

In the present study, we employed an integrative research strategy combining network pharmacology analysis, molecular docking, molecular dynamics simulations, and *in vivo* experimental validation to systematically investigate the mechanisms by which HXJGCC facilitates tibial fracture healing. Special attention was given to signaling pathways related to angiogenesis and tissue regeneration. By integrating computational predictions with biological verification in a rabbit tibial fracture model, this study aimed to clarify the molecular targets and regulatory pathways through which HXJGCC enhances fracture repair, while further exploring the potential interactions among vascular, neural, and osteogenic processes during bone regeneration. The proposed mechanistic framework is illustrated in Figure 1.

## Materials and methods

### Study design, reporting standards, and ethics approval

This study employed an integrative multi-level research strategy combining network pharmacology prediction, molecular docking, molecular dynamics simulation, and *in vivo* experimental validation to investigate the mechanisms by which HXJGCC promotes tibial fracture healing. The overall experimental workflow was designed to identify bioactive compounds, predict key molecular targets, and validate angiogenesis-associated mechanisms during fracture repair (16). All animal experiments were approved by the Animal Ethics Committee of Guizhou University of Traditional Chinese Medicine (Approval No. 20230148) and conducted in accordance with the ARRIVE reporting guidelines and institutional animal welfare regulations. Investigators performing histological evaluation, qRT-PCR analysis, and image quantification were blinded to treatment allocation (17).

Forty healthy New Zealand white rabbits (2.0 ± 0.5 kg; equal numbers of males and females) were obtained from an accredited laboratory animal center. Animals were acclimatized for 7 days under controlled environmental conditions (temperature 22 ± 2 °C, humidity 55 ± 10%, 12-h light/dark cycle) with free access to food and water. All animals were included in the experimental analysis, and no animals were excluded during the study period. Each animal was considered an independent biological replicate for subsequent analyses. Rabbits were randomly assigned using SPSS v26.0 randomization procedures into four groups (*n* = 10 per group): Model group; HXJGCC low-dose group; HXJGCC medium-dose group; HXJGCC high-dose group. Humane endpoints were predefined, and animals were euthanized using an overdose of sodium pentobarbital when required.

### Composition, preparation, and administration of HXJGCC

HXJGCC was supplied by the First Affiliated Hospital of Guizhou University of Traditional Chinese Medicine and produced under Good Manufacturing Practice (GMP) standards. All herbal components were authenticated by pharmacognosy specialists and complied with the Chinese Pharmacopoeia (2020 edition). This preparation comprises ten herbal ingredients, namely Gusuibu, Xuduan, Zirantong, Tubiechong, Danshen, Tougucao, Sumu, Sanqi, Bingpian, and Yanhusuo. The botanical names, medicinal parts, and dosages of these herbs are detailed in Table 1. All crude drugs were pulverized into fine powder, homogenized, and encapsulated in hard gelatin capsules. Prior to administration, the capsule powder was suspended in sterile 0.9% physiological saline to obtain a uniform suspension. Rabbit dosages were determined using body surface area conversion from the clinical human dose. The equivalent doses were set as: Low dose: 0.5 g/kg/day; Medium dose: 1.0 g/kg/day; High dose: 2.0 g/kg/day. The suspension was administered once daily by oral gavage (5 mL/kg) for 5 consecutive weeks. Animals in the model group received an equivalent volume of saline.

**Table 1:** Ten botanical drugs of HXJGCC.

Chinese name	Name of pubishing	Used parts	Weight (g)
Gusuibu	Rhizoma Drynariae	Rhizome	12
Xuduan	Radix Dipsaci	Root	12
Zirantong	Pyritum	Mineral (Iron Pyrite)	9
Tubiechong	Eupolyphaga seu Steleophaga	Female Insect (Dried Body)	9
Danshen	Radix et Rhizoma Salviae Miltiorrhizae	Root and Rhizome	12
Tougucao	Herba Speranskiae Tuberculatae	Whole Plant	15
Sumu	Lignum Sappan	Heartwood	9
Sanqi	Radix et Rhizoma Notoginseng	Root and Rhizome	12
Bingpian	Borneolum Syntheticum	Synthetic Crystal	5
Yanhusuo	Rhizoma Corydalis	Tuber	9

### Establishment of the tibial segmental defect model and perioperative management

A rabbit tibial segmental defect model was established to evaluate the effects of HXJGCC on fracture repair. Animals were anesthetized using isoflurane inhalation anesthesia. After shaving and sterilization, a 1.5-cm longitudinal incision was made on the anterolateral aspect of the tibia, approximately 1.5 cm distal to the tibial plateau and 1.5 cm lateral to the anterior tibial crest. The tibialis anterior muscle was bluntly separated to expose the tibial shaft. Under continuous saline irrigation to prevent thermal injury, a 3-mm mid-shaft segmental bone defect was created using a precision wire saw. Internal stabilization was achieved using a 1.0-mm intramedullary Kirschner wire, ensuring alignment and mechanical stability of the tibia. The wound was then closed in layers. Postoperative care included butorphanol for analgesia and penicillin for infection prophylaxis. Rabbits were allowed free cage activity after surgery. HXJGCC administration began on postoperative day 2 according to the dosing protocol described above.

This model reliably reproduces the dynamic biological processes occurring during fracture repair, including vascular reconstruction, callus formation, and tissue remodeling, and is widely used for investigating mechanisms related to bone regeneration and angiogenesis.

### Tissue collection and experimental timeline

To evaluate fracture repair dynamically, animals were euthanized at three postoperative time points: Week 1 (early inflammatory and angiogenic phase), Week 3 (active callus formation and vascular maturation), Week 5 (remodeling phase). At each time point, three to four rabbits per group were randomly selected for tissue harvesting. Bone samples containing the defect region and adjacent bone (approximately 0.5–1 cm on each side) were collected. The right tibia was immediately isolated and processed according to downstream experimental requirements (18). Samples were allocated as follows: histological analysis; qRT-PCR analysis.

### Reagents, instruments, and quality control

All reagents and instruments used for qRT-PCR and histological experiments, including manufacturers and catalog numbers, are listed in Supplementary material 1. Key reagents included RNA extraction reagent U7431 (Suzhou U7 Biotechnology), RT SuperMix G3337, and 2 × SYBR Green Master Mix G3326 (Wuhan Servicebio). RNA concentration and purity were assessed using a microvolume spectrophotometer (Thermo Fisher, UL61010-1). All experiments were conducted strictly according to standardized protocols to ensure reproducibility and data reliability.

### TCM theoretical basis and functional grouping of HXJGCC

HXJGCC is formulated based on the core TCM pathogenesis of fracture and impaired bone healing, characterized by liver–kidney deficiency, qi and blood insufficiency, and blood stasis obstructing the collaterals (19). The prescription follows the principle of reinforcing deficiency while eliminating stasis. Drynariae Rhizoma, Salviae Miltiorrhizae Radix, and Dipsaci Radix act as monarch herbs to tonify the liver and kidney and promote fracture repair; Pyritum, Eupolyphaga sinensis, and Panax notoginseng serve as minister herbs to activate blood circulation and facilitate callus formation; Sappan Lignum, Gaultheria yunnanensis, and Corydalis Rhizoma function as assistant herbs to unblock collaterals, relieve pain, and improve the local microenvironment; Borneolum acts as the guide herb to direct drug actions to the lesion site.

Based on core therapeutic functions, HXJGCC herbs were classified into four functional groups: stasis-removing and bone-strengthening, blood-activating and fracture-repairing, collateral-unblocking and analgesic, and meridian-guiding and synergistic-enhancing groups.

### Analysis of TCM properties, flavors, and meridian tropism

Information on the properties, flavors, and meridian tropism of each HXJGCC component was collected from the Chinese Pharmacopoeia. “Herb–meridian,” “herb–four properties,” and “herb–five flavors” networks were constructed to systematically analyze the overall TCM characteristics of the formula and interpret its potential therapeutic basis for fracture healing from a TCM perspective (20).

### Screening of potential active compounds and target prediction

Candidate active compounds of HXJGCC were retrieved from the TCMSP database and supplemented by literature mining (21). For herbs with limited database information (e.g., Sappan Lignum, Pyritum, Eupolyphaga sinensis), additional data were obtained from the HERB database. Canonical SMILES structures were acquired from PubChem. Oral bioavailability (OB ≥ 30%) and drug-likeness (DL ≥ 0.18) were used as primary screening criteria. Potential targets were predicted using SwissTargetPrediction (species: *Homo sapiens*; probability > 0.1) (22)and standardized to HGNC gene symbols via UniProt (23). Compounds lacking SMILES structures or predictable targets were excluded.

### Identification of tibial fracture–related targets

Tibial fracture–related targets were retrieved from the GeneCards database (relevance score ≥ 3) and the OMIM database using the keyword “Tibial Fracture.” After UniProt standardization to HGNC symbols, targets were merged and deduplicated to generate the disease-related gene set.

### Construction and analysis of PPI networks

Intersection targets between each functional group (and the whole formula) and tibial fracture–related genes were identified using Venny 2.1.0 and visualized via an online bioinformatics platform. These targets were imported into the STRING database to construct protein–protein interaction (PPI) networks (species: *Homo sapiens*; confidence score > 0.4) (24). Network visualization and topological analysis were performed using Cytoscape 3.10.3 (25).

### GO annotation and KEGG pathway enrichment analysis

Gene Ontology (GO) functional annotation—including biological processes (BP), cellular components (CC), and molecular functions (MF)—and KEGG pathway enrichment analyses were conducted using the DAVID database (26). Terms with *p* < 0.05 were considered statistically significant. For the whole formula, the top 20 KEGG pathways ranked by significance were selected for presentation.

### Construction of the “formula–functional group–herb–target–disease” network

Based on the above analyses, Cytoscape 3.10.3 was used to construct networks linking herbs, active compounds, and key targets for each functional group and the whole formula. Topological parameters were calculated, and nodes with degree, betweenness, and closeness values above the median were identified as core compounds and core targets for subsequent molecular docking and dynamics analyses.

### Molecular docking analysis

Representative core compounds (hesperidin, cnidiosin A_qt, kaempferol, cnidiosin, quercetin, and tetrahydrotanshinone) were docked with key targets (BCL2, IL6, EGFR, JUN, AKT1, and STAT3). Ligand structures were obtained from PubChem, and protein structures were downloaded from the Protein Data Bank and filtered via UniProt. Protein preparation was performed using AutoDockTools 1.5.7 (27). Docking was conducted with AutoDock Vina, with 10 independent runs per complex. The lowest binding energy conformation was selected for analysis and visualized using PyMOL.

### Molecular dynamics simulations

Selected compound–target complexes were subjected to 100-ns molecular dynamics simulations using YASARA with the AMBER14 force field (28). Systems were solvated in a cubic periodic box (≥10 Å) with TIP3P water and 0.15 M NaCl. After protonation at pH 7.4 and energy minimization, equilibration was performed under NVT (310 K) and NPT (1 atm, 310 K) ensembles. Long-range electrostatics were treated using the PME method, with a 2-fs integration step. RMSD, RMSF, hydrogen bond number, radius of gyration, and binding energy changes were analyzed.

### Quantitative real-time PCR

Bone tissue encompassing the defect region was homogenized on ice, and total RNA was extracted using U7431 reagent. After genomic DNA removal, cDNA was synthesized using RT SuperMix G3337. qRT-PCR was performed using 2 × SYBR Green Master Mix G3326, with GAPDH as the internal control. Expression levels of AKT1, STAT3, IL6, and EGFR were quantified. Each sample was analyzed in three biological replicates, three technical replicates, and relative expression was calculated using the 2^−ΔΔCt method.

### Histological analysis and vascular quantification

Right tibiae were fixed in 4% paraformaldehyde, decalcified, dehydrated, paraffin-embedded, and sectioned at 4 μm for H&E staining. Images were acquired at 200× or 400× magnification. Three non-overlapping regions of interest within the defect/callus area were selected per animal. Vascular density (vessels·mm^−2^) and vascular area (μm^2^/ROI) were quantified using ImageJ (29). All analyses were performed in a blinded manner.

### Statistical analysis

Statistical analyses were conducted using GraphPad Prism v9.3. Data normality and variance homogeneity were assessed using the Shapiro–Wilk and Brown–Forsythe/Levene tests, respectively. One-way ANOVA followed by Tukey’s or Dunnett’s *post hoc* tests was applied for single-time-point comparisons, while two-way ANOVA was used for multi-time-point analyses. qRT-PCR data were analyzed using ΔCt or log2-transformed values. Results are presented as mean ± SD, and *p* < 0.05 was considered statistically significant.

## Results

### Characteristics of the four properties, five flavors, and meridian tropism of HXJGCC

Based on the TCM attribute curation and network construction strategy described in the Methods, we first systematically analyzed the four properties, five flavors, and meridian tropism of HXJGCC to clarify its overall prescription orientation and theoretical therapeutic implications, thereby providing a foundation for subsequent component screening and mechanistic analyses (30).

HXJGCC comprises multiple herbs with distinct therapeutic attributes. Analysis of the herb–four properties network revealed a predominance of warm and mildly warm herbs, supplemented by cold and neutral components, reflecting a balanced formulation characterized by “warming with regulation,” which is considered favorable for promoting blood circulation, unblocking collaterals, and facilitating bone repair. The herb–five flavors network demonstrated that bitter and pungent flavors predominated, with auxiliary sweet components contributing harmonization, highlighting therapeutic features of blood stasis removal, analgesia, and bone strengthening—consistent with the TCM principle that fracture pathology is “rooted in stasis and manifested as pain.”

Analysis of the herb–meridian network showed that HXJGCC primarily targets the Liver and Kidney meridians, with secondary involvement of the Heart meridian, emphasizing the synergistic roles of tendon regulation, bone nourishment, and vascular modulation. Collectively, these results indicate that HXJGCC exhibits a coherent and well-defined TCM theoretical orientation supporting its functions in removing stasis, strengthening bone, relieving pain, and promoting fracture repair.

### Screening of potential active compounds and therapeutic targets of HXJGCC

Following clarification of the overall TCM characteristics, we systematically screened and integrated potential active compounds and corresponding targets across different functional groups to explore the material basis underlying HXJGCC-mediated fracture repair. A total of 209 non-redundant candidate active compounds were identified, including 91 from the stasis-removing and bone-strengthening group, 14 from the blood-activating and fracture-repairing group, 113 from the collateral-unblocking and analgesic group, and 3 from the meridian-guiding and synergistic-enhancing group.

**Figure 2:**
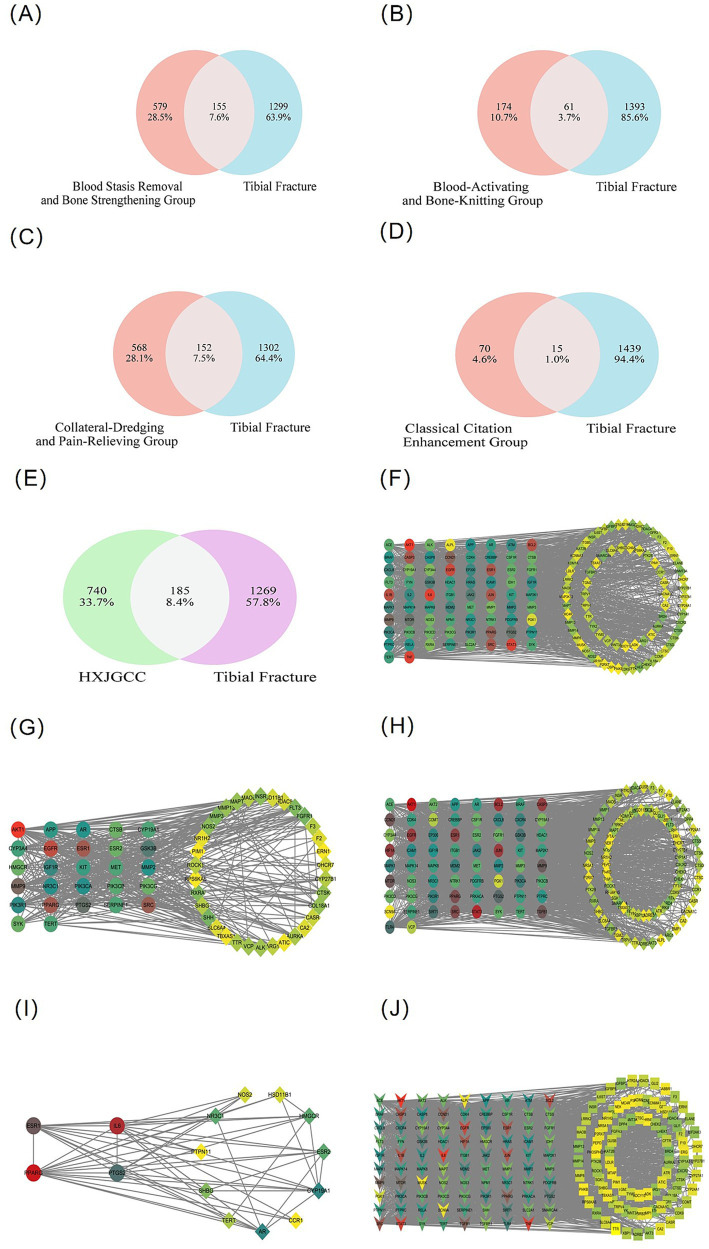
Target intersection and network topology analysis of HXJGCC functional modules related to tibial fracture. **(A–D)** Venn diagrams showing overlapping targets between predicted targets of four TCM functional groups and tibial fracture–related genes. **(E)** Intersection between targets of the whole HXJGCC formula and tibial fracture–related genes. **(F–J)** Protein–protein interaction (PPI) networks after topological screening. Key targets were identified based on degree, betweenness centrality, and closeness centrality values above the median. Node size represents topological importance and edges represent protein–protein interactions.

Using SwissTargetPrediction, 926 non-redundant potential targets were predicted (31). As shown in Figures 2A–D, the numbers of overlapping targets between each functional group and tibial fracture–related genes were 155, 61, 152, and 15, respectively, while 185 shared targets were identified for the whole formula (Figure 2E).

Topological analysis further identified key targets with degree, betweenness, and closeness centrality values above the median. Accordingly, 74, 27, 66, 4, and 90 key targets were obtained for the respective functional groups and the whole formula (Figures 2F–J). Notably, the stasis-removing and bone-strengthening group and the collateral-unblocking and analgesic group contained the largest numbers of key targets, suggesting that these modules may represent the principal functional contributors to HXJGCC-mediated fracture repair.

### Functional group–specific “herb–compound–target” networks and pathway characteristics

**Figure 3:**
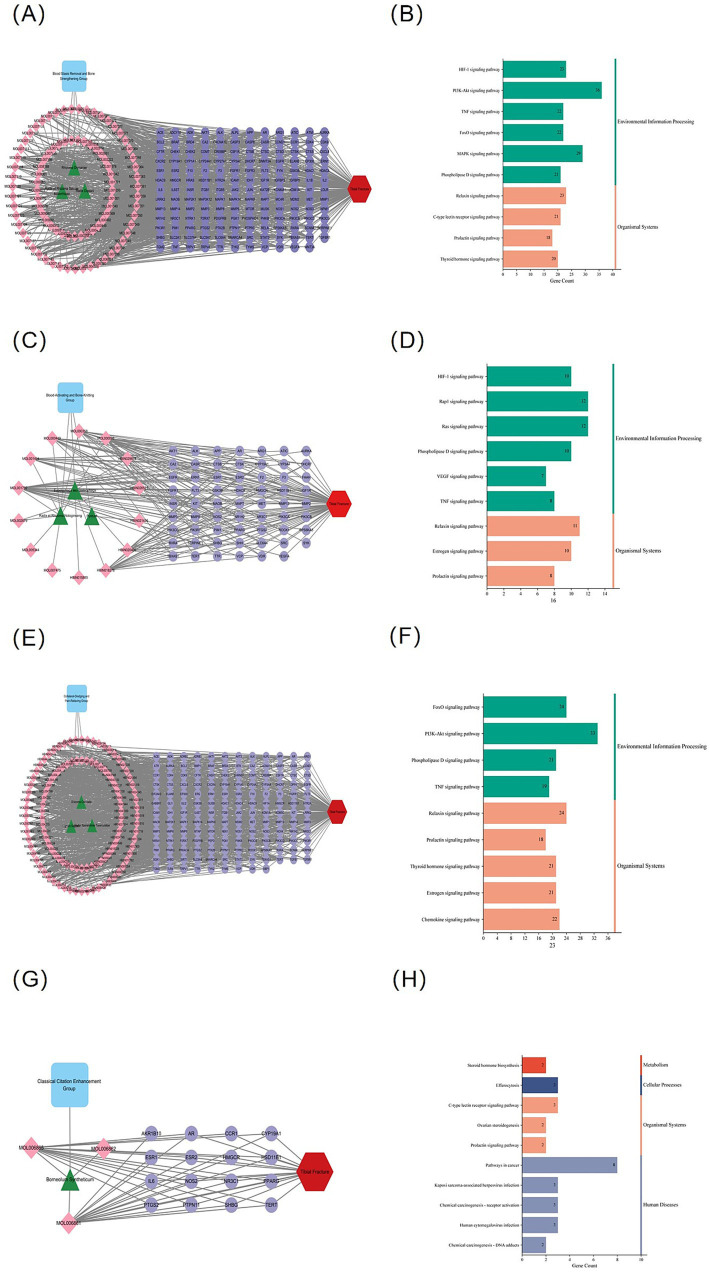
Herb–compound–target networks and KEGG pathway enrichment analysis of HXJGCC functional groups. **(A,C,E,G)** Herb–active compound–key target networks for four TCM functional groups within HXJGCC. **(B,D,F,H)** KEGG pathway enrichment analysis of key targets from each functional group. The top enriched pathways include HIF-1, PI3K–Akt, Relaxin, TNF, and FoxO signaling pathways.

To further elucidate the molecular features of each functional group, we constructed “herb–active compound–key target” networks and performed KEGG pathway enrichment analyses. As shown in Figures 3A,B, key targets of the stasis-removing and bone-strengthening group were mainly enriched in the HIF-1, PI3K–Akt, Relaxin, TNF, and C-type lectin receptor signaling pathways, indicating potential involvement in inflammation regulation, angiogenesis, and cell survival. Key targets of the blood-activating and fracture-repairing group were enriched in PI3K–Akt, Relaxin, HIF-1, Rap1, and Estrogen signaling pathways (Figures 3C,D). The collateral-unblocking and analgesic group primarily involved HIF-1, Relaxin, FoxO, PI3K–Akt, and Prolactin signaling pathways (Figures 3E,F), whereas the meridian-guiding group was mainly associated with Efferocytosis and Steroid hormone biosynthesis pathways (Figures 3G,H).

Overall, these results demonstrate both functional differentiation across groups and substantial pathway convergence, particularly within HIF-1, PI3K–Akt, and Relaxin signaling, suggesting coordinated and synergistic regulatory mechanisms (32, 33).

### PPI network and functional enrichment analysis of key targets of the whole formula

**Figure 4:**
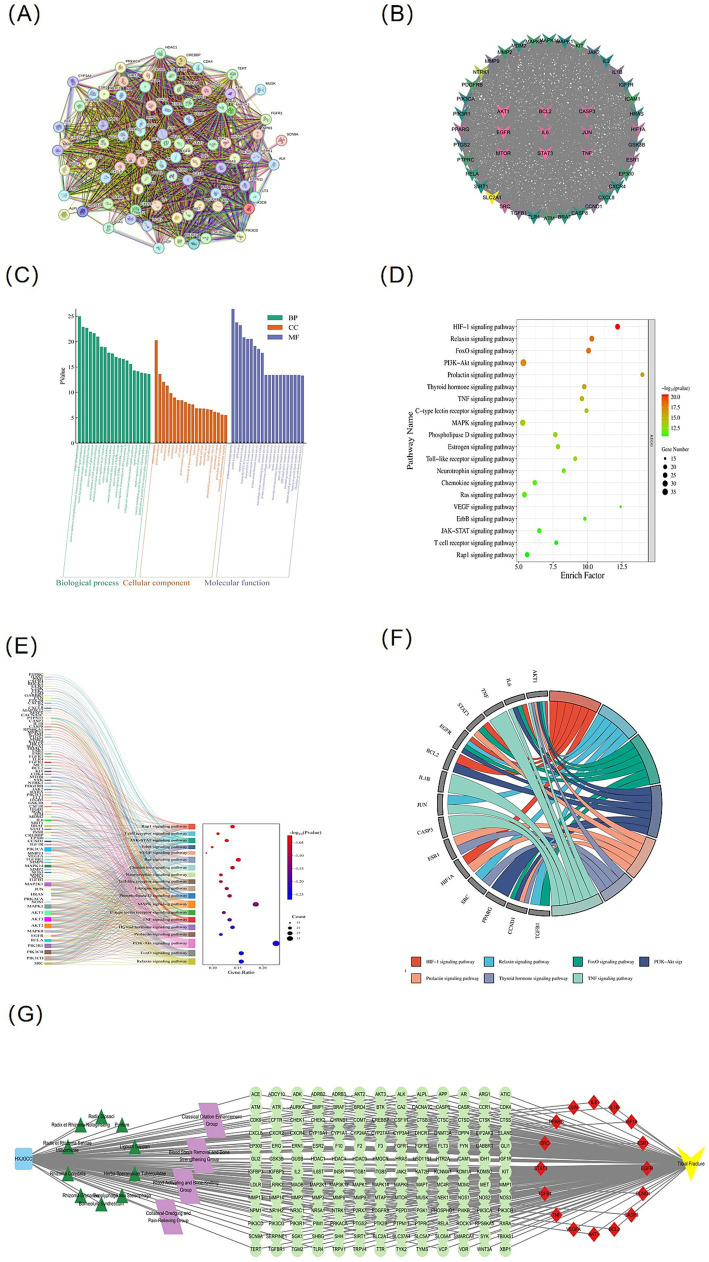
Network analysis and functional enrichment of key targets associated with HXJGCC-mediated fracture repair. **(A)** STRING-based protein–protein interaction (PPI) network of 90 key targets. **(B)** Core target subnetwork screened by degree centrality (degree ≥ 46). **(C)** Gene Ontology (GO) enrichment analysis. **(D)** KEGG pathway enrichment analysis. **(E)** Pathway–gene interaction network. **(F)** Chord diagram showing relationships between hub genes and enriched pathways. **(G)** Integrated “formula–functional group–herb–target–disease” network illustrating the multi-component and multi-target characteristics of HXJGCC.

To elucidate the cooperative mechanisms of HXJGCC at the systems level, a STRING-based protein–protein interaction (PPI) network was constructed for the 90 key targets of the whole formula. The resulting network comprised 90 nodes and 1,876 edges, indicating high connectivity (Figure 4A). Based on degree centrality (≥46), 15 core targets were identified (Figure 4B), For specific degree values, please refer to Supplementary material 2, most of which were closely associated with inflammation, cell survival, and angiogenesis.

GO enrichment analysis (Figure 4C) showed that key targets were primarily involved in inflammatory responses, cellular stress responses, apoptosis regulation, and angiogenesis. KEGG analysis (Figure 4D) further highlighted significant enrichment in the HIF-1, PI3K–Akt, TNF, Relaxin, and FoxO signaling pathways. In the pathway–gene network (Figures 4E,F), AKT1, IL6, EGFR, BCL2, JUN, and STAT3 appeared repeatedly across multiple pathways, exhibiting pronounced hub characteristics. The top 20 results from the specific GO and KEGG enrichment analysis are presented in Supplementary Table 1. The integrated “formula–functional group–herb–target–disease” network (Figure 4G) systematically illustrates how distinct functional groups cooperatively regulate fracture-related pathological processes through shared and complementary targets.

### Molecular docking analysis of core targets

**Figure 5:**
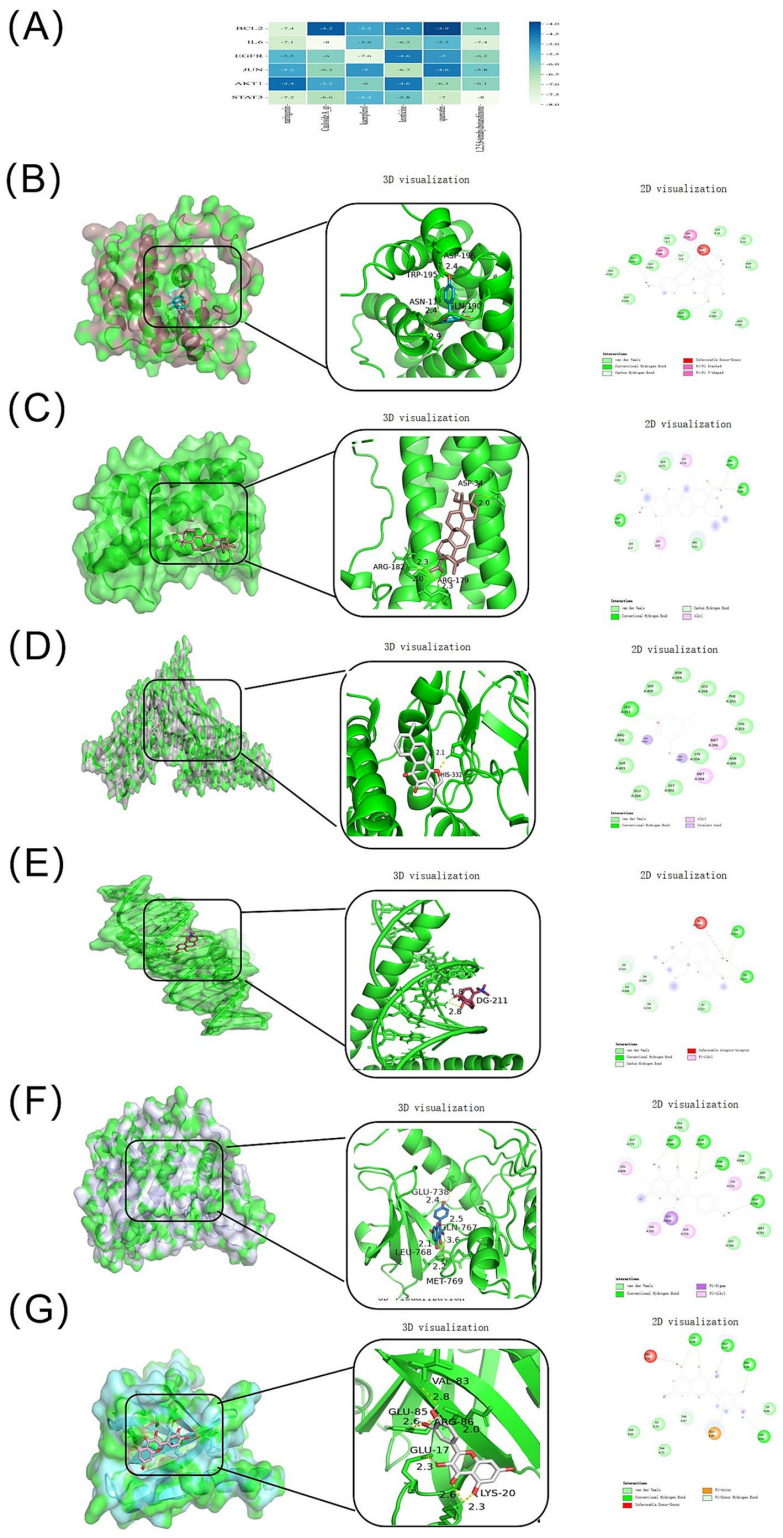
Molecular docking analysis between representative active compounds and core targets of HXJGCC. **(A)** Heatmap of docking binding energies. **(B–G)** Representative docking conformations of ligand–protein complexes: **(B)** AKT1–naringenin; **(C)** IL6–Cauloside A_qt; **(D)** STAT3–tetrahydrotanshinone; **(E)** JUN–leonticine; **(F)** EGFR–kaempferol; **(G)** BCL2–quercetin. Binding energies ≤ −5.0 kcal/mol indicate favorable interactions.

To validate potential interactions between key active compounds and core targets, molecular docking analyses were performed for six representative compounds against six core targets. As shown in Figure 5A, most ligand–protein complexes exhibited binding energies ≤ −5.0 kcal/mol, indicating favorable binding affinity. Among them, Cauloside A_qt–IL6 and tetrahydrotanshinone–STAT3 displayed the lowest binding energies (both −8.0 kcal/mol).

Three-dimensional and two-dimensional interaction analyses (Figures 5B–G) demonstrated that all ligands stably occupied the active pockets of target proteins through hydrogen bonding and hydrophobic interactions, supporting a multi-component–multi-target synergistic mechanism at the molecular level.

### Molecular dynamics simulation validation

**Figure 6:**
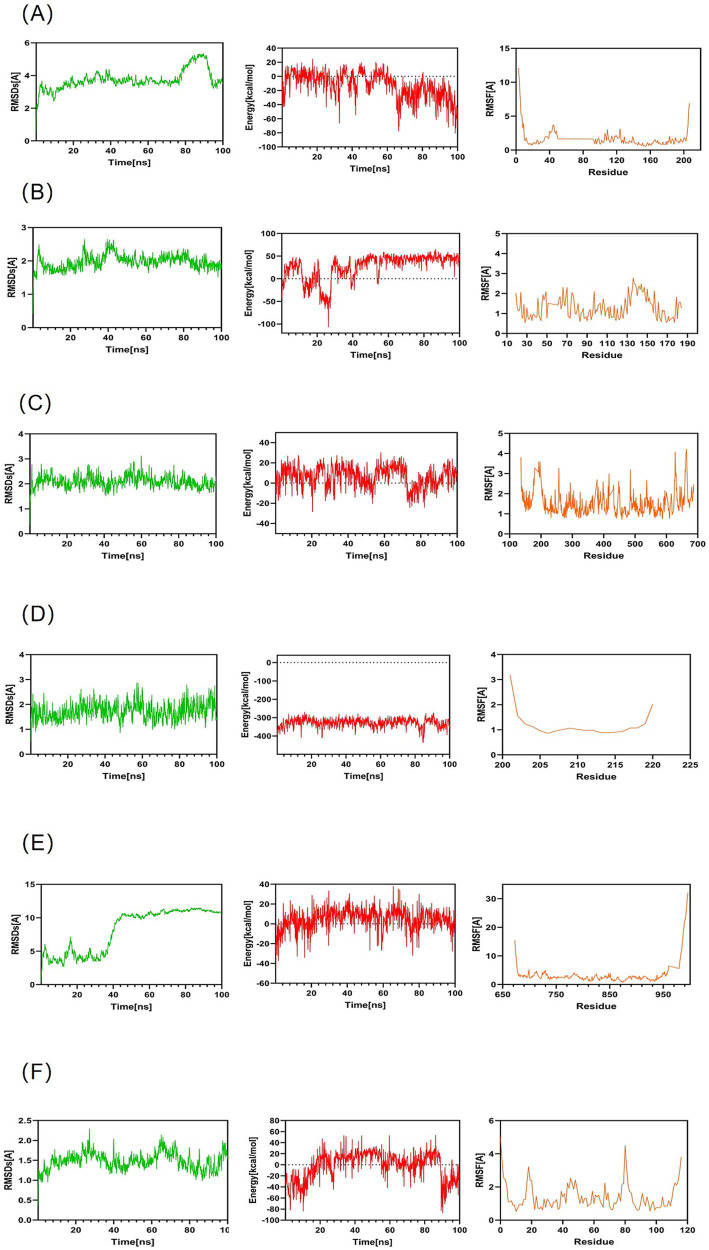
Molecular dynamics simulation of ligand–target complexes. 100-ns molecular dynamics simulations were performed for six representative complexes. Panels **(A–F)** show time-dependent profiles of root mean square deviation (RMSD), binding energy, and root mean square fluctuation (RMSF), indicating the stability of protein–ligand interactions.

To further assess docking stability, 100-ns molecular dynamics simulations were conducted for the six ligand–target complexes. The molecular dynamics simulation parameters can be found in Supplementary Table 2. As shown in Figure 6A–F, most complexes rapidly reached RMSD equilibrium with limited fluctuations, maintained consistently negative binding energies, and exhibited low RMSF values within binding regions, indicating favorable dynamic stability. These findings were highly consistent with the docking results and further strengthened the reliability of the predicted compound–target interactions.

### Regulatory effects of HXJGCC on key gene expression

**Figure 7:**
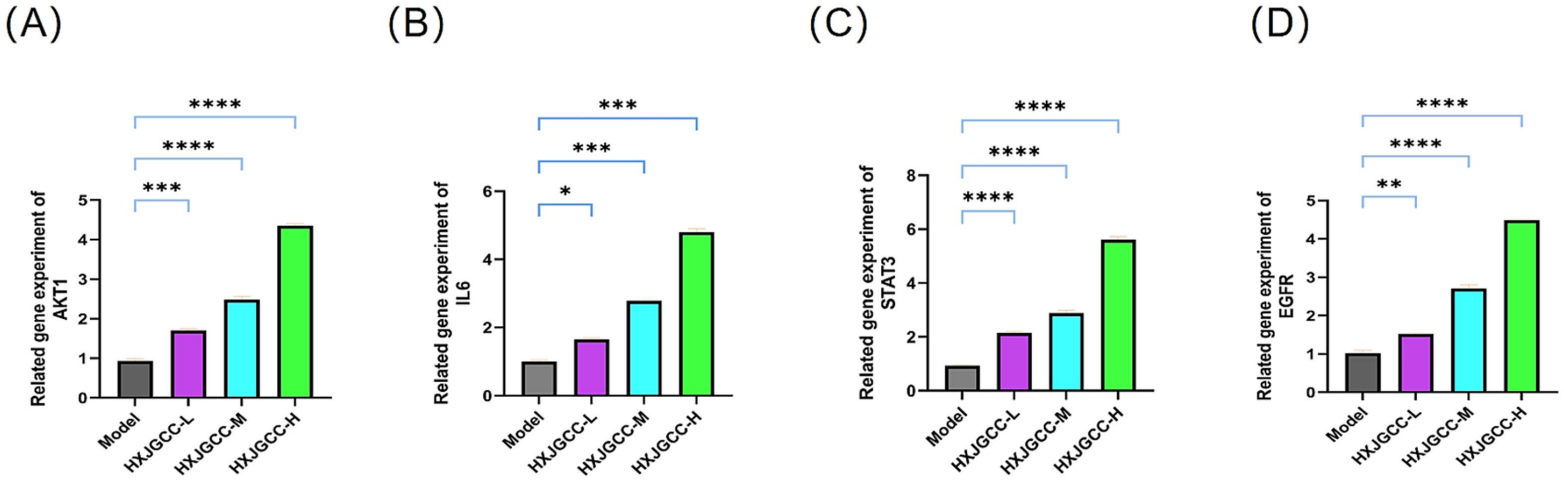
Effects of HXJGCC on the expression of core hub genes *in vivo*. **(A–D)** Relative mRNA expression levels of AKT1, IL6, STAT3, and EGFR in bone tissue measured by qRT-PCR. Data are presented as mean ± SD (*n* = 3 biological replicates). Statistical analysis was performed using one-way ANOVA followed by Tukey’s *post hoc* test. Significance levels:*p < 0.05, **p < 0.01, ***p < 0.001, ****p < 0.0001 vs. model group.

To validate the network and simulation predictions *in vivo*, the effects of HXJGCC on AKT1, IL6, STAT3, and EGFR expression were examined. As shown in Figures 7A–D, all HXJGCC-treated groups exhibited significantly increased expression of these genes compared with the model group, with most showing dose-dependent trends. Notably, expression of AKT1, STAT3, and EGFR in the high-dose group reached extremely significant levels (*p* < 0.0001), indicating robust transcriptional activation of core hub genes.

### Effects of HXJGCC on angiogenesis in bone tissue

**Figure 8:**
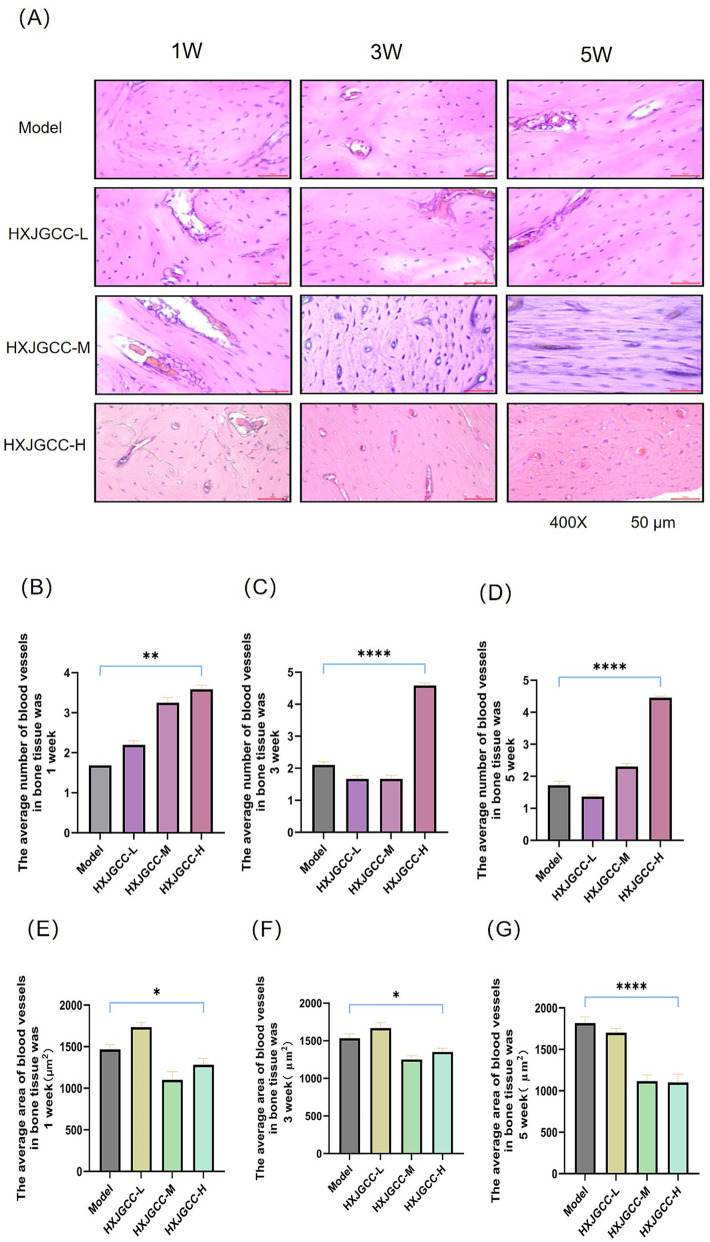
HXJGCC enhances angiogenesis during tibial fracture repair. **(A)** Representative H&E-stained histological sections of bone tissue showing vascular structures (arrows) at 1, 3, and 5 weeks after treatment. **(B–D)** Quantification of vessel number per field. **(E–G)** Quantification of vascular area per field. Histomorphometric analysis was performed using ImageJ software. Data are expressed as mean ± SD (*n* = 3–4 animals per group). Statistical analysis was conducted using two-way ANOVA followed by Tukey’s post hoc test. *p < 0.05, **p < 0.01, ***p < 0.001 vs. model group.

Given the prominent enrichment of angiogenesis-related pathways, histological analyses were performed to evaluate vascular changes in bone tissue. As shown in Figure 8A, sparse and disorganized vasculature was observed in the model group, whereas HXJGCC treatment resulted in progressively increased vascular density and improved structural integrity over time. Quantitative analysis (Figures 8B–G) demonstrated that HXJGCC significantly enhanced vessel number and dynamically optimized vascular area distribution. Notably, the high-dose group exhibited structural refinement rather than simple vascular enlargement at later stages.

Collectively, these results indicate that HXJGCC promotes angiogenesis and vascular maturation, thereby dynamically improving the local blood supply microenvironment and facilitating fracture repair.

## Discussion

Fracture healing is a highly coordinated regenerative process involving inflammatory regulation, vascular reconstruction, osteogenic differentiation, and tissue remodeling (34). Among these biological events, angiogenesis represents a critical determinant of successful bone regeneration, as newly formed microvessels provide oxygen, nutrients, and progenitor cells required for callus formation and skeletal repair (35). Insufficient vascularization within the fracture microenvironment has been widely recognized as an important cause of delayed union or nonunion (36). Therefore, therapeutic strategies that enhance vascular regeneration are considered promising approaches for improving fracture healing outcomes (37).

In the present study, we investigated the mechanisms by which HXJGCC promotes tibial fracture repair using an integrative strategy combining network pharmacology, molecular docking, molecular dynamics simulation, and *in vivo* experimental validation. Our findings collectively suggest that HXJGCC accelerates fracture healing primarily by enhancing angiogenesis and improving the local microvascular microenvironment (38), while potentially coordinating downstream osteogenic and neuro-regulatory processes.

Network pharmacology analysis revealed that HXJGCC contains multiple bioactive compounds capable of interacting with numerous molecular targets associated with fracture repair (39). Protein–protein interaction analysis identified AKT1, STAT3, BCL2, IL6, EGFR, and JUN as key hub targets within the regulatory network (40, 41). These molecules have been widely implicated in tissue regeneration, inflammatory regulation, and vascular remodeling. Enrichment analysis further demonstrated that these targets were significantly involved in several signaling pathways closely associated with angiogenesis and tissue repair, including HIF-1, PI3K–Akt, Relaxin, TNF, and FoxO signaling pathways (42). These pathways play essential roles in regulating endothelial cell proliferation, vascular remodeling, and the adaptive response of tissues to hypoxic microenvironments during bone repair (43).

Among these pathways, HIF-1 signaling represents a central regulator of hypoxia-induced angiogenesis during fracture healing. Following bone injury, local hypoxia rapidly activates HIF-1–dependent transcriptional programs that stimulate vascular growth and recruit regenerative cells to the fracture site (44). Activation of the PI3K–Akt pathway further promotes endothelial cell survival, migration, and vascular stabilization, thereby supporting the formation of functional microvascular networks within the callus tissue. In addition, inflammatory cytokine signaling pathways such as TNF and IL-6–related cascades participate in coordinating early inflammatory responses with subsequent vascular and osteogenic activities. The enrichment of these pathways in our network analysis suggests that HXJGCC may regulate fracture repair through integrated signaling networks centered on angiogenic activation (45).

To further validate these computational predictions, molecular docking and molecular dynamics simulations were performed to evaluate the binding stability between representative HXJGCC compounds and core protein targets. The results demonstrated favorable binding affinities between key compounds such as quercetin, kaempferol, hesperidin, and tetrahydrotanshinone and several hub targets, including AKT1, STAT3, IL6, and EGFR (46). Molecular dynamics simulations confirmed the stability of these compound–target complexes during the 100-ns simulation period, suggesting that the identified interactions are structurally stable and may contribute to the pharmacological effects of HXJGCC. These findings support the concept that the therapeutic effects of traditional Chinese medicine formulations are mediated through multi-component, multi-target regulatory networks rather than a single molecular pathway.

*In vivo* experiments further provided biological evidence supporting the angiogenesis-centered mechanism suggested by the computational analyses. Histological examination of the fracture region revealed that HXJGCC treatment significantly increased vascular density and microvascular organization within the callus tissue compared with the untreated model group. Enhanced vascularization within the defect region is likely to improve oxygen delivery, nutrient supply, and the recruitment of osteogenic precursor cells, thereby facilitating the progression from early inflammatory stages to active callus formation (3). These findings are consistent with previous studies demonstrating that improved vascular reconstruction plays a fundamental role in promoting bone regeneration.

At the molecular level, qRT-PCR analysis showed that HXJGCC treatment significantly upregulated the expression of AKT1, STAT3, IL6, and EGFR in bone tissue during the fracture healing process. These genes are closely associated with angiogenesis, inflammatory regulation, and cellular proliferation. Activation of the AKT1-related signaling network is known to promote endothelial cell survival and vascular growth, whereas STAT3 signaling has been implicated in coordinating inflammatory responses and tissue regeneration (47). The upregulation of these genes suggests that HXJGCC may activate a complex regulatory network that supports vascular remodeling and tissue repair within the fracture microenvironment.

Although the present study primarily focused on angiogenesis, increasing evidence suggests that fracture healing involves coordinated interactions among multiple biological systems. Sensory nerve fibers are known to innervate bone tissue and may influence vascular and osteogenic responses through the release of neuropeptides and neurotrophic factors (48, 49). Previous studies have proposed the concept of neuro–vascular–bone interactions in skeletal regeneration, suggesting that neural signaling may modulate endothelial activity and bone remodeling. In the context of our findings, enhanced vascularization induced by HXJGCC may provide a favorable microenvironment that indirectly supports these broader regenerative processes. However, it should be noted that direct neural markers were not evaluated in the present study, and therefore the involvement of neural regulation should be considered a potential rather than confirmed mechanism.

The results of this study also support the therapeutic principles of traditional Chinese medicine traumatology. According to TCM theory, traumatic injury disrupts the circulation of qi and blood within meridians, leading to impaired nourishment of bone and surrounding tissues. Herbal formulations that promote blood circulation and remove blood stasis are therefore commonly used to restore tissue regeneration (50). Modern pharmacological studies have increasingly demonstrated that many of these herbal medicines possess angiogenic, anti-inflammatory, and osteogenic activities, providing a biological explanation for their clinical efficacy (51). HXJGCC contains multiple herbal components with reported pharmacological effects related to vascular regeneration and tissue repair, which may collectively contribute to the angiogenesis-promoting activity observed in this study.

Despite these findings, several limitations should be acknowledged. First, although network pharmacology analysis predicted multiple signaling pathways associated with fracture repair, experimental validation in the present study focused on a limited number of representative genes. Additional studies examining other pathway components may further clarify the regulatory mechanisms of HXJGCC. Second, the present study primarily evaluated vascular changes using histological methods, and more specific endothelial markers could provide additional evidence for angiogenic activity. Third, while potential neuro–vascular–bone interactions were discussed based on existing literature, direct evaluation of neural markers was not performed. Fourth, although protein-level validation was not performed, the consistency between network pharmacology prediction, molecular docking stability, and transcriptional upregulation provides convergent multi-level evidence supporting the involvement of angiogenesis-related pathways. Future studies incorporating neurogenic factors, endothelial markers, osteogenic indicators, and protein-level validation will be necessary to further elucidate the integrated regulatory network underlying fracture repair.

In summary, this study demonstrates that HXJGCC promotes tibial fracture healing primarily by enhancing angiogenesis and improving the microvascular environment within the fracture region. Through the activation of key regulatory pathways including HIF-1 and PI3K–Akt signaling, HXJGCC may coordinate inflammatory responses, vascular remodeling, and tissue regeneration during the bone healing process. These findings provide mechanistic insights into the multi-target pharmacological actions of HXJGCC and highlight angiogenesis-driven repair mechanisms as a potential therapeutic strategy for improving fracture healing outcomes.

## Conclusion

In conclusion, this study demonstrates that HXJGCC accelerates tibial fracture healing primarily through angiogenesis-driven repair mechanisms. Integrative analyses combining network pharmacology, molecular docking, molecular dynamics simulations, and *in vivo* validation identified key regulatory targets, including AKT1, STAT3, IL6, and EGFR, and highlighted angiogenesis-related pathways such as HIF-1 and PI3K–Akt signaling. Experimental results further confirmed that HXJGCC significantly enhances vascular density and improves the microvascular microenvironment within the fracture region. These findings suggest that HXJGCC promotes fracture repair mainly by stimulating angiogenic responses and supporting tissue regeneration, while potentially facilitating broader neuro–vascular–bone interactions during bone healing. Collectively, this work provides mechanistic insight into the multi-target therapeutic effects of HXJGCC and highlights angiogenesis-centered therapeutic regulation as a promising strategy for enhancing fracture healing.

## Data Availability

The raw data supporting the conclusions of this article will be made available by the authors without undue reservation.

## Glossary

HXJGCC: HuoXue JieGu Compound Capsules
AKT1: murine thymoma viral oncogene homolog 1
STAT3: Signal Transducer and Activator of Transcription 3
PI3K: Akt Phosphatidylinositol 3-kinase/Protein kinase B signaling pathway
IL6: Interleukin-6
CC: Cellular Component
MAPK: Mitogen-Activated Protein Kinase
MF: Molecular Function
DL: Drug-Likeness
PPI: Protein–Protein Interaction
GO: Gene Ontology
KEGG: Kyoto Encyclopedia of Genes and Genomes
MD: Molecular Dynamics
qRT-PCR: Quantitative Real-Time Polymerase Chain Reaction
HE: Hematoxylin and Eosin (staining)
RMSD: Root-Mean-Square Deviation
BP: Biological Process
EGFR: Epidermal Growth Factor Receptor
HIF-1α/VEGF: Hypoxia-Inducible Factor 1-alpha/Vascular Endothelial Growth Factor
TCMSP: Traditional Chinese Medicine Systems Pharmacology Database and Analysis Platform
OB: Oral Bioavailability
RMSF: Root-Mean-Square Fluctuation
GMP: Good Manufacturing Practice.
