# Supplementary material for: Huoxue Jiegu compound capsule accelerates tibial fracture healing via angiogenesis-driven repair mechanisms
Source: Front Med (Lausanne). 2026 May 11;13:1810673. doi: 10.3389/fmed.2026.1810673 (PMC13199261; doi:10.3389/fmed.2026.1810673)
Supplement: Supplementary file 1 [file Supplementary_file_1.docx]

**Supplementary Material 1**: Experimental Reagents, Instruments, and Specific Procedures

1.**Experimental Reagents and Instruments**

- 1. qPCR Reagents

RNA extraction reagent (Suzhou U7 Bio-Tech Co., Ltd., U7431), RNA EX Buffer (Shenzhen Mohong Technology Co., Ltd., B9025), sterile enzyme-free water (Beijing Solabio Technology Co., Ltd., R1600), SweScript All-in-One RT SuperMix for qPCR (Wuhan Savier Biotechnology Co., Ltd., G3337). 2× Universal Blue SYBR Green qPCR Master Mix (Wuhan Savier Biotechnology Co., Ltd., G3326), Isopropanol (Sinopharm Chemical Reagent Co., Ltd., 80109218), Anhydrous Ethanol (Sinopharm Chemical Reagent Co., Ltd., 10009218).

- 1. Histopathology staining reagents

HE staining kit (Beijing Solarbio Science & Technology Co., Ltd., G1121), 4% tissue/cell fixative (Beijing Solarbio Science & Technology Co., Ltd., P1110), paraffin embedding medium (Beijing Solarbio Science & Technology Co., Ltd., YA0012), Neutral Gum (Beijing Solarbio Technology Co., Ltd., G8590), 1× PBS Buffer (pH 7.2–7.4) (Beijing Solarbio Technology Co., Ltd., P1020), Tween-20 (Beijing Solarbio Technology Co., Ltd., T8220), EDTA Decalcification Solution (Slow Decalcification) (Wuhan Savier Biotechnology Co., Ltd., G1105-500ML).

1. **Experimental Instruments**

Microcentrifuge (Beijing Dalongxingchuang Experimental Instrument Co., Ltd., D1012U), Grinder (China Xiaomayi Instrument Equipment Co., Ltd., MY-4), Ultrasonicator (Wuxi Yuansheng Intelligent Technology Co., Ltd., 20KLS-1200B), Vortex mixer (Haimen Qilinbel Instrument Manufacturing Co., Ltd., VORTEX-5), Electronic analytical balance (Shanghai Lichenbangxi Instrument Technology Co., Ltd., LC-FA1004), Micro-volume spectrophotometer (Thermo Fisher Scientific, USA, UL61010-1), High-speed refrigerated centrifuge (Thermo Fisher Scientific, USA, LR56495), Class II Biosafety Cabinet (Shanghai Xiniu Technology Co., Ltd., BHC-100011A2), -80°C Ultra-Low Temperature Freezer (Hangzhou Xiling Electric Co., Ltd., DL-278-86), Vertical Pressure Steam Sterilizer (Jiangsu Dengguan Medical Equipment Co., Ltd., DGLS-50B), Digital Display Orbital Shaker (Haimen Qilinbei Instrument Manufacturing Co., Ltd., China, SCI-O180-S), Forced-Air Circulating Drying Oven (Shanghai Titan Technology Co., Ltd., FO-50), Refrigerator (Skyworth Electric Co., Ltd., BCD-625WKPS), Induction Cooker (Zhejiang Supor Co., Ltd., C22-IA815), Fume Hood (Suzhou Antai Air Technology Co., Ltd., BHC-100012B2), Paraffin Embedding Machine (Aitree Medical Technology (Hubei) Co., Ltd., ATR-BML50), Paraffin Microtome (Leica Microsystems GmbH, Germany, Modell RM2235), Tissue Stretcher and Dryer (Xiamen Zhiwei Instruments Co., Ltd., China, ATR-TKH10), Biological Optical Microscope (Shanghai Cewei Optoelectronic Technology Co., Ltd., LW370LT), Laboratory Pure Water System (Shanghai Xiniu Technology Co., Ltd., UPST-40L), Constant Temperature Water Bath (Changzhou SuiRui Instrument Co., Ltd., HH-2), Ice Maker (Ningbo Polar Bear Electric Co., Ltd., BJ-Z9BYT).

1. **Fluorescent Quantitative PCRSpecific Procedures**

(1) Lysate bone tissue with lysis buffer. Add 2/5 volume of RNase-free H₂O (200 μL water per 500 μL Adazol) to the lysis buffer, vigorously vortex to mix, and let stand at room temperature for 5 min. For larger sample volumes (approximately 50 mg), extend the room temperature incubation time to 10–15 min. 3. Centrifuge at 12,000 rpm for 15 min at room temperature.

(2) After centrifugation, the supernatant (containing RNA) separates from the pellet (containing proteins, DNA, polysaccharides, and other impurities). Carefully transfer the supernatant to a new centrifuge tube.

(3) Add an equal volume of isopropanol, mix by inverting, and let stand at room temperature for 10 min.

(4) Centrifuge at 12,000 rpm for 10 min at room temperature. A white precipitate is typically visible; carefully discard the supernatant;

(5) Add 1 mL 75% ethanol (prepared with RNase-free ddH₂O) for washing. Vortex for 15 sec to resuspend the pellet, then invert several times;

(6) Centrifuge at 12,000 rpm for 3 min at room temperature. Carefully remove the supernatant;

(7) Repeat steps (5) and (6) for one additional wash. Carefully remove all supernatant. To minimize impurity carryover, ensure complete removal of supernatant. After discarding most supernatant, briefly tap the tube to settle residual liquid to the bottom. Use a 200 μL pipette tip to aspirate all remaining liquid, retaining the white RNA pellet adhering to the tube bottom and walls;

(8) Air-dry at room temperature for approximately 1 minute. Add an appropriate volume of RNase-free ddH₂O to dissolve the pellet. Vortex at room temperature for 3 minutes (or repeatedly pipette the pellet at the bottom and sides of the tube to aid dissolution) to ensure complete dissolution of the RNA pellet. The extracted RNA product can be aliquoted and stored long-term at -80°C. Short-term storage is possible between -30°C and -15°C.

(9) gDNA digestion: Prepare the reaction mixture in RNase-free PCR tubes according to Table 1. Perform all reaction mixture preparation steps on ice. Gently mix by pipetting. Incubate at 42°C for 2 min.

**4.Hematoxylin and eosin staining Specific Procedures**

(1) Sample Decalcification: Decalcify samples according to the decalcification solution instructions before proceeding with subsequent testing.

(2) Preparation of Tissue Paraffin Specimens and Sections: Place formaldehyde-fixed tissues into tissue embedding cassettes, mark with pencil, then rinse with water for 15 minutes. then sequentially immerse in a gradient of alcohol solutions (anhydrous ethanol I, anhydrous ethanol II, 95% ethanol I, 95% ethanol II, 85% ethanol, 75% ethanol I, 75% ethanol II, 50% ethanol I, 50% ethanol II) for 30 minutes each, from highest to lowest concentration. After complete dehydration, transfer to xylene I for 1 hour immersion, followed by 1 hour in Xylene II. Transfer the embedding cassette to the paraffin embedding machine and immerse in liquid paraffin at 60°C for 2 hours. After thorough saturation, add clean liquid paraffin for embedding. For sectioning, pre-cool paraffin specimens overnight at 4°C. After removal, securely mount vertically on the microtome. Cut 4μm paraffin sections onto the front side of a slide. Tilt the slide at 45° into 40°C warm water, utilizing surface tension to flatten the section. Place the section into a slide holder for later use.

(3) Hydration of Sections: Place the prepared paraffin sections in a 65°C section warmer for 45 minutes to remove excess paraffin. Subsequently, transfer the paraffin sections to Xylene I and Xylene II (30 minutes each), Anhydrous Ethanol I and Anhydrous Ethanol II (15 minutes each), 95% ethanol I and 95% ethanol II (15 min each), 85% ethanol (15 min), 75% ethanol I and 75% ethanol II (15 min each), 50% ethanol I and 50% ethanol II (15 min each). Finally, immerse the sections in distilled water for 30 min to hydrate.

(4) Hematoxylin staining for 3 seconds, followed by water rinsing (perovskite solution dark-staining for 10 minutes, then water rinsing);

(5) Eosin staining for 10 seconds, followed by water rinsing (Schiff's solution dark-staining for 20 minutes, then water rinsing, hematoxylin staining for 3 seconds, then water rinsing);

(6) Gradient dehydration with ethanol solutions: 75% ethanol → 85% ethanol → 95% ethanol → anhydrous ethanol II → anhydrous ethanol I (5 seconds per step);

(7) Xylene clearing (twice), total duration approximately 10 min;

(8) Routine mounting: Wipe excess xylene from the periphery of the section, promptly add an appropriate amount of neutral resin, then cover with a coverslip to seal.

(9) Microscopic examination: Randomly photograph three non-overlapping fields of view under light microscopy (×400, ×200).
