# Supplementary material for: Huoxue Jiegu compound capsule accelerates tibial fracture healing via angiogenesis-driven repair mechanisms
Source: Front Med (Lausanne). 2026 May 11;13:1810673. doi: 10.3389/fmed.2026.1810673 (PMC13199261; doi:10.3389/fmed.2026.1810673)
Supplement: Supplementary file 2 [file Supplementary_file_2.docx]

**Supplementary Material 2**

The core targets of the top 15 Degree values in HXJGCC.

| Key targets | Degree |
| --- | --- |
| AKT1 | 126 |
| IL6 | 122 |
| TNF | 119 |
| STAT3 | 117 |
| EGFR | 112 |
| BCL2 | 109 |
| IL1B | 107 |
| JUN | 107 |
| CASP3 | 104 |
| ESR1 | 104 |
| HIF1A | 103 |
| SRC | 101 |
| PPARG | 96 |
| CCND1 | 96 |
| TGFB1 | 94 |

|  |
| --- |

Degree: In the cell landscape, the degree of a node is a key attribute that represents the number of edges connected to it. In general, the higher the degree value, indicating that the node plays a more important or core role in the network.
